# Natural Variability of Kozak Sequences Correlates with Function in a Zebrafish Model

**DOI:** 10.1371/journal.pone.0108475

**Published:** 2014-09-23

**Authors:** Steven J. Grzegorski, Estelle F. Chiari, Amy Robbins, Phillip E. Kish, Alon Kahana

**Affiliations:** Ophthalmology and Visual Sciences, Kellogg Eye Center, University of Michigan, Ann Arbor, Michigan, United States of America; University Zürich, Switzerland

## Abstract

In eukaryotes, targeting the small ribosomal subunit to the mRNA transcript requires a Kozak sequence at the translation initiation site. Despite the critical importance of the Kozak sequence to regulation of gene expression, there have been no correlation studies between its natural variance and efficiency of translation. Combining bioinformatics analysis with molecular biology techniques, and using zebrafish as a test case, we identify Kozak sequences based on their natural variance and characterize their function *in* *vivo*. Our data reveal that while the canonical Kozak sequence is efficient, in zebrafish it is neither the most common nor the most efficient translation initiation sequence. Rather, the most frequent natural variation of the Kozak sequence is almost twice as efficient. We conclude that the canonical Kozak sequence is a poor predictor of translation efficiency in different model organisms. Furthermore, our results provide an experimental approach to testing and optimizing an important tool for molecular biology.

## Introduction

The central dogma of molecular biology defines the transfer of information from DNA to protein through an RNA intermediate [1]. The irreversible transfer of information from RNA to protein relies on ribosomal translation. Both prokaryotic and eukaryotic translation progress through initiation, elongation and termination phases. For successful initiation, the small ribosomal subunit must successfully bind to the translational start site and recruit the large ribosomal subunit to form a functional ribosome. In prokaryotes, the small ribosomal subunit targets the initiation site through the base pairing of the 16s rRNA and the Shine-Dalgarno sequence on the mRNA transcript. Originally characterized in 1974, the Shine-Dalgarno sequence is a consensus sequence (AGGAGGU) generally found immediately upstream of the translational start site [2].

In eukaryotes, targeting the small ribosomal subunit to the mRNA transcript is complicated through the addition of required protein: protein interactions [3]. However, characterization of nucleotide sequences surrounding known translational start sites revealed a DNA consensus sequence (5′-GCCRCC**ATG**G-3′) suggesting a conserved role in translational efficiency [4]. This consensus sequence, now known as the Kozak consensus sequence, has become increasingly valuable as a tool for improving artificial expression constructs. Nevertheless, since the initial characterization in 1987, little has been done to functionally characterize the natural sequences in vertebrates. Furthermore, although the Kozak consensus sequence has been incorporated in a multitude of expression studies, no *in* *vivo* validation has been done to correlate the Kozak sequence with functional expression levels. (In this manuscript, we utilize the term “Kozak sequence” to refer to the sequence encoding the translation initiation site, and the term “canonical Kozak sequence” to refer to the original consensus sequence described by Kozak in 1987).

This study uses an approach that combines bioinformatics with molecular biology to identify Kozak sequences across multiple model systems and test specific sequences for translational efficiency using the zebrafish model system. Using this approach, we identified an optimal Kozak sequence for translational efficiency in zebrafish, and demonstrated a correlation between natural variance and translational efficiency. A similar approach can be taken in any organism with a sequenced genome, potentially improving the molecular tools at our disposal.

## Methods

### Fish Husbandry

All protocols were approved by the University Committee for the Use and Care of Animals (UCUCA protocol 3628), in compliance with federal and international norms and regulations. Zebrafish (*Danio rerio*) were maintained on an AB background. Adult fish were mated using standard techniques. Embryos were collected into E3 embryo media and staged as previously described [5].

### Sequence Analysis

Sequences were extracted from the NCBI nucleotide data base in March 2013. Briefly, a search was done at www.ncbi.nlm.nih.gov/nuccore for a specific taxonomy (e.g. txid7955 for *Danio rerio*) and results were filtered for refseq sequences. Results were saved to a file using the website features. Sequences with the NM prefix were extracted from the resulting text document using Hex Fiend (http://ridiculousfish.com/hexfiend/). Sequences were then imported into Microsoft Excel for final tabulations.

### Polymerase Chain Reaction (PCR)-mediated cloning of Kozak sequences

Kozak sequences were cloned in front of the eGFP reporter gene using a 2-step PCR technique that utilized the CS2p+EGFPbgl2 plasmid as template (courtesy of David Turner, University of Michigan; http://sitemaker.umich.edu/dlturner.vectors/files/cs2p_plus_EGFP_bgl2.txt). In the first step, the plasmid was linearized with *HindIII*, followed by PCR (Taq) using a forward primer that placed the canonical or an alternate Kozak sequence between an SP6 promoter and the eGFP gene, and a reverse T3 primer (Table 1). The forward primers were: Frequent-EGFP, Rare-EGFP, Middle-EGFP, Worst-EGFP and Kozak-EGFP (Table 1). Following the first-step PCR, fragments were gel purified and a second PCR was performed using SP6 and reverse T3 primers (Table 1). DNA fragments were precipitated before resuspension in DNase/RNase free water.

**Table 1:** Primer sequences.

Primer	Sequence
Reverse T3	5′-ATTAACCCTCACTAAAG
SP6	5′-ATTTAGGTGACACTATAG
Frequent-EGFP	5′-ATTTAGGTGACACTATAGAAGCAAACATGGCGGTGAGCAAGGGCGAGGAG
Rare-EGFP	5′-ATTTAGGTGACACTATAGAACTTTCTATGCTCGTGAGCAAGGGCGAGGAG
Middle-EGFP	5′-ATTTAGGTGACACTATAGAAGCAGTCATGGAGGTGAGCAAGGGCGAGGAG
Worst-EGFP	5′-ATTTAGGTGACACTATAGAACGTTGTATGCTGGTGAGCAAGGGCGAGGAG
Kozak-EGFP	5′-ATTTAGGTGACACTATAGAAGCCACCATGGCGGTGAGCAAGGGCGAGGAG
EGFP qPCR F	5′-CAGAAGAACGGCATCAAGGTG
EGFP qPCR R	5′-GGACTGGGTGCTCAGGTAGTG
Elf1a qPCR F	5′-CTCCTCTTGGTCGCTTTGCT
Elf1a qPCR R	5′-GCCTTCTGTGCAGACTTTGTGA

### RNA transcription and injection solution preparation

Using 250 ng of purified PCR product, mRNA was synthesized using the SP6 mMessage mMachine kit (Ambion) and LiCl precipitated according to the Ambion kit manufacturer protocol. Successful transcription was verified using a Nanodrop spectrophotometer (Thermo Scientific). Stock mRNA solutions were diluted to 100 ng/uL. Zebrafish embryo injection solutions were made in 0.1 M KCl to a final mRNA concentration of 50 ng/uL with phenol red added to assist with injection verification. Approximately 1 nL of solution was injected directly into the cell of 1-cell embryos using a Narishige IM 300 Microinjector.

### Quantitative real time reverse transcription PCR (qRT-PCR)

Groups of 25 embryos were collected at 24 hours post fertilization (hpf), homogenized in Trizol (Life Technologies, Inc.) and processed according to Peterson and Freeman [6]. eGFP mRNA was quantified using qRT-PCR normalized against elongation factor 1a (*elf1a*) as an endogenous normalization control. Primers used were EGFP qPCR F, EGFP qPCR R; Elf1a qPCR F, and Elf1a qPCR R (Table 1).

### eGFP Fluorescence quantification

Groups of 25 embryos (post mRNA injection) were collected at ∼24 hpf and homogenized in 100 mM tris (pH 7.5) +1% Triton X-100 and left on ice for 20 minutes. After 20 min centrifugation at 4°C to pellet the insoluble material, 2 uL were removed and used to quantify protein content using the Pierce BCA kit (Thermo Scientific). The remaining solubilized embryos in Tris solution were diluted to equivalent protein concentrations. A Qubit fluorometer (Life Technologies) was used to measure eGFP fluorescence (in arbitrary units) relative to a positive control of 1∶500 dilution of Goat-anti mouse 488 (Thermo Scientific #35502).

Fluorometer values were normalized using the mRNA ratio [eGFP/elf1a] to correct for the small variations in eGFP mRNA that was injected into embryos (Figure S1). Statistical significance was assessed using an unpaired *t*-test and GraphPad Prism software.

## Results

### Bioinformatic Analysis of Vertebrate Translational Start Sites

**Figure 1:**
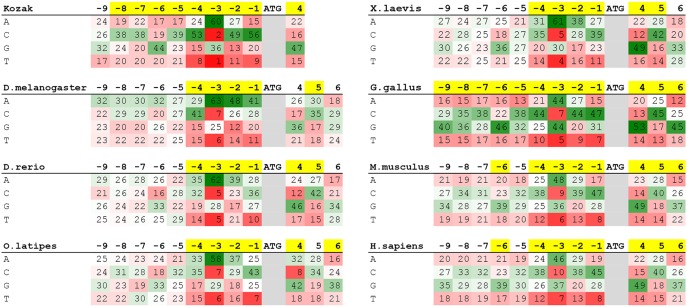
Quantitative sequence analysis reveals positive selection for nucleotides surrounding translational start sites. −9 to +6 nucleotide frequencies (%) are presented for the consensus Kozak sequence and 7 model species. Yellow indicates positions with statistically significant selection (p<0.03). Frequencies are color coded on a 3 color gradient from 0% (red) to 25% (white) to >50% (green).

Sequence information for 7 well studied species was downloaded from the NCBI refseq database (Table 2). Only sequences with an NM accession prefix were used, indicating validated mRNA sequences. After identifying ATG start sites, individual nucleotide frequency for positions −9 to +6 were calculated and are presented in figure 1. In both mice and human, Swindell et al. showed that the nucleotide frequency from positions −2000 to +200 surrounding transcription start sites is approximately even at 25% for each base pair [7]. Extending this assumption to all species, a chi square test was done for each individual base pair using the neutral frequency matrix of 25∶25∶25∶25 as expected. Based on this test, observed nucleic acid frequencies at each position that significantly differed from the expected matrix were colored yellow in figure 1.

**Table 2:** Bioinformatic data sets and consensus Kozak sequences.

Data Set	# of sequences	Consensus
Original Kozak (1987)	667	5′-CCGCCACCATGG
*D. melanogaster*	26265	5′-CAAAATGgC
*D. rerio*	12926	5′-AAACATGGC
*O. latipes*	380	5′-CAACATGGcG
*X. laevis*	10397	5′-CAACATGGC
*G. gallus*	4326	5′-GGCGCCGCCATGGCG
*M. musculus*	24433	5′-GcCACCATGGCG
*H. sapiens*	32526	5′-GcCACCATGGCG

Based on this frequency data, “Ideal” translation initiation consensus sequences were identified (Table 2) in which nucleotide selection frequency was statistically significantly above 25% (p<.05). The results confirmed the importance across all analyzed species of the −3 residue, and further provide potentially important species-specific modifications to the canonical Kozak sequence. Notably, the size of the significant portion of the consensus sequence varies considerably across the groups with *gallus gallus* having the largest “Kozak” sequence. Additionally, the identified consensus sequences include only the more prevalent nucleotide at each position even though some positions in the dataset may have 2 base pairs with similar frequencies.

### Breakdown of the Zebrafish Transcriptome

Using the 12,926 predicted mRNA sequences from the *Danio rerio* genome, positions −4 through +5 were identified as statistically significant by the frequency-based selection criteria. Considering these 6 variable nucleotides, 4,096 unique sequences are possible. Of these, 2,403 appear at least once in the data set. Notably, 2,165 of these unique sequences occur in 0.1% or less of the data set, while the remaining 238 sequences (or approximately 10%) account for almost 50% of translational start sites in the predicted mRNA sequences (see List S1).

**Figure 2:**
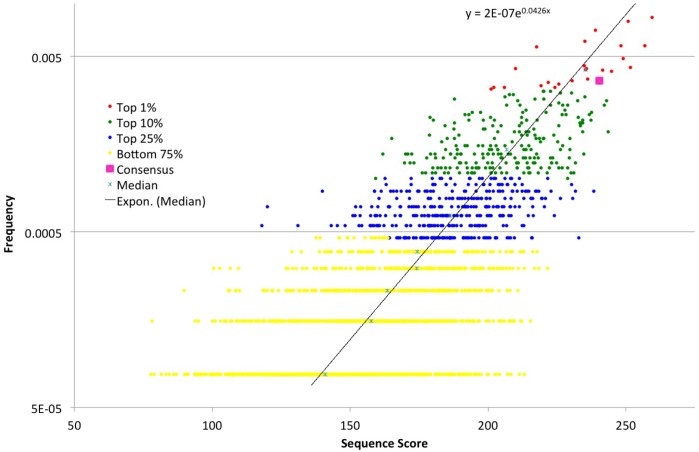
Natural frequency (y axis) correlates with the comparative sequence-selection score. Unique translation initiation sequences demonstrate a relationship between natural frequency and the comparative score. The comparative scoring system was developed to relate unique sequences to their individual nucleotide frequencies relative to the entire population (see text). The exponential function was calculated using the median score from each frequency group, revealing a modest correlation between comparative score and natural frequency. The points are color-coded based on natural frequency. The consensus/canonical Kozak sequence is marked in pink.

Using the zebrafish-specific criteria in figure 1, a comparative scoring system was devised to identify sequences for further downstream *in* *vivo* testing. For each of the 2,165 unique sequences, a score was assigned by summing the frequency of each nucleotide in the sequence based on the numbers in figure 1. For example, the most frequent sequence (AAAC**ATG**GC) received respective scores of 35+62+39+36+46+42 for a total of 260. Scores for individual sequences were then graphed against the overall sequence frequency in the population (Figure 2). Higher sequence scores indicate increased selection for individual nucleotides within the sequence. Despite the broad range of scores and the weight given to low scores, a log graph of the scores reveals that beneficial sequences (i.e. high scores) do not necessarily correspond to increased frequency in the population, but high natural frequencies appear to correlate with higher relative scores. Notably, the highest score (AAAC**ATG**GC; 260) represented the most frequent translational start site in zebrafish (108/12926) (see List S1).

### *In Vivo* validation of translational start sites

Building on the visualization provided by figure 2, it was decided to test the *in* *vivo* relevance of the scoring system and attempt to identify a link between *in* *vivo* translational efficiency and assigned score. Furthermore, given the widespread use of the canonical Kozak sequence in molecular biology and genetic engineering research, understanding the translational impact of species-specific variations around the translational start site would also be of significant value.

**Figure 3:**
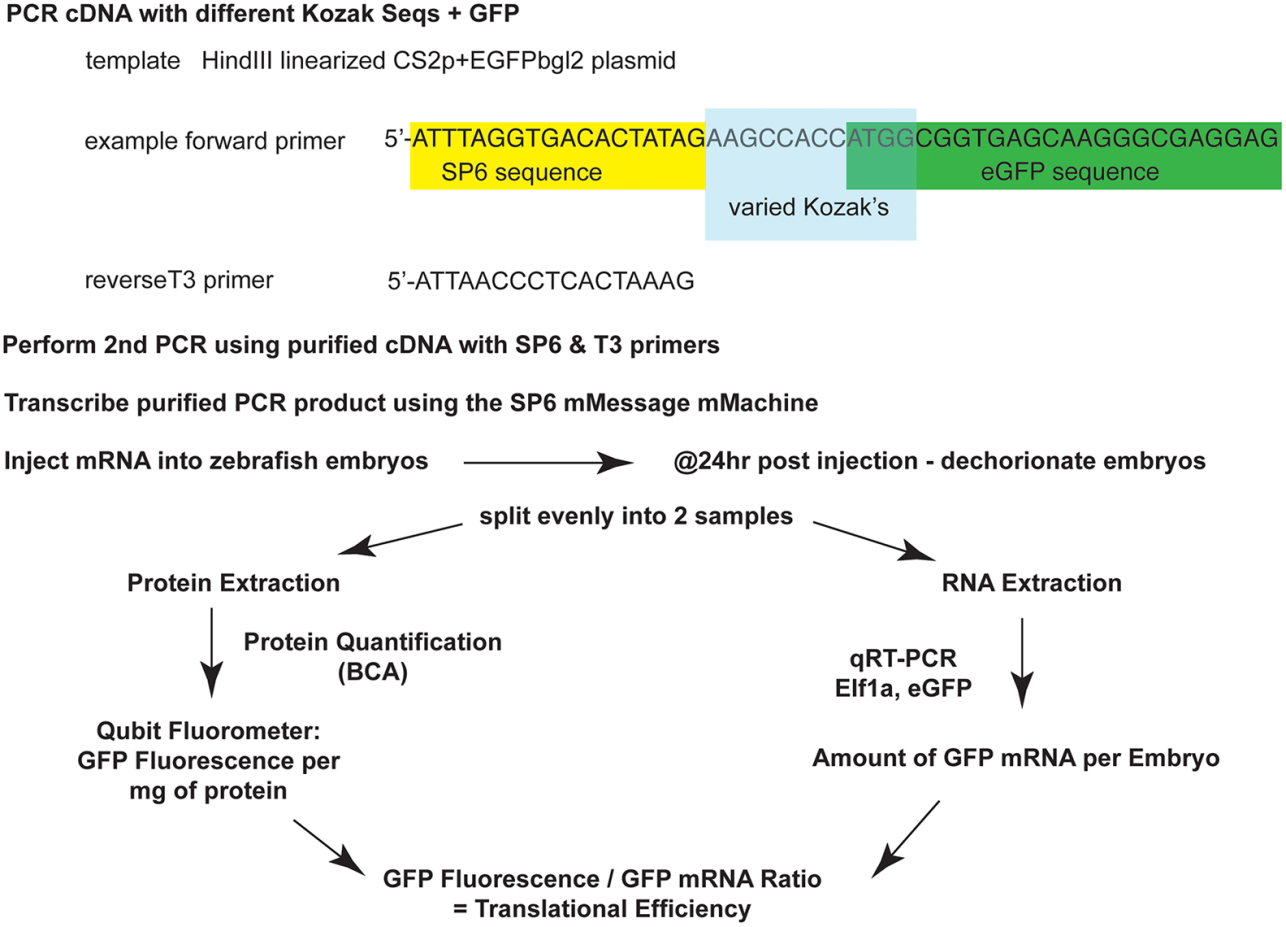
Schematic of a PCR-based method to correlate Kozak sequence with translation efficiency. Following PCR reactions with nested primers, the purified PCR product is transcribed and the RNA quantified and injected into 50 embryos per RNA product. At 24 hours post injection, the embryos are divided into 2 groups of 25 embryos – one group is used for RNA extraction, to validate the amount of RNA that was injected, and the other group is used for protein extraction, to assess translation. *elf1a* was used as an endogenous normalization control for qRT-PCR. Fluorescence from eGFP is normalized to total protein (see text and Figure S1).

**Figure 4:**
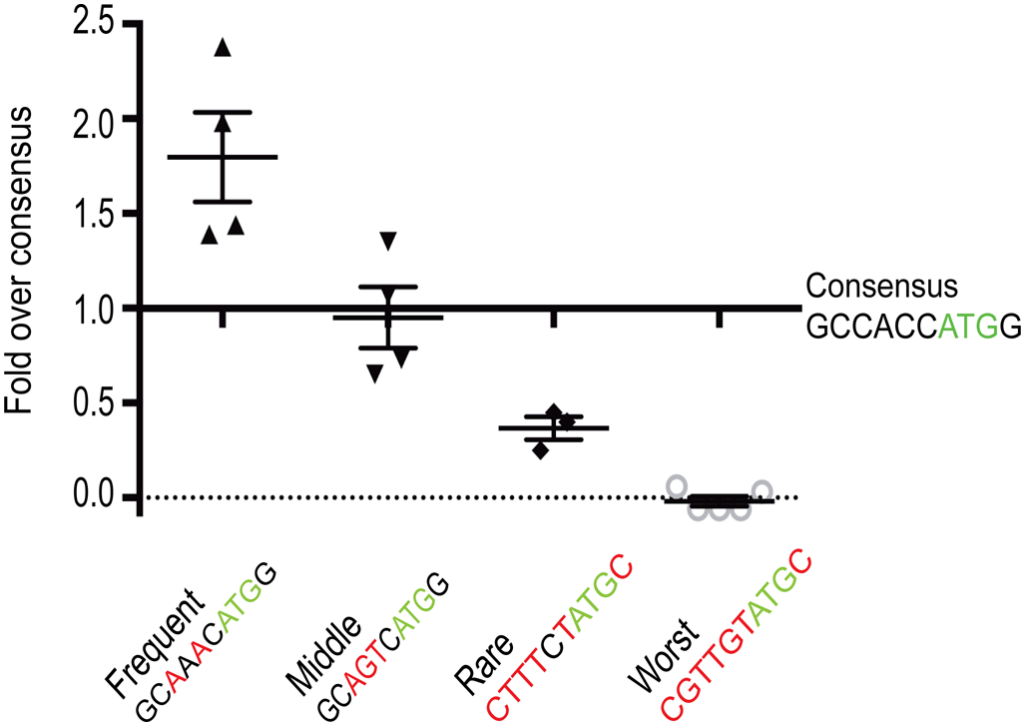
Nucleotide sequence surrounding translational start sites modifies expression levels. Translational efficiency is measured by fluorescent expression relative to the canonical/consensus Kozak sequence. All samples are statistically different from each other (p<0.04) except for Kozak vs. Middle. Each condition represents 4 replicates of 25 embryos each. One replicate from each condition was performed at the same time (i.e. one set) in order to achieve maximal consistency, and the replicate for the consensus Kozak sequence from that set was used to normalize the other conditions. Therefore, the consensus Kozak relative expression level is defined as 1 for each set of replicates, and that number lacks error bars. The fold over/under consensus has an order of magnitude range, providing no sign of assay saturation.

To test whether translational efficiency correlated with natural sequence variation scores, a PCR based cloning method was used to generate a set of eGFP-encoding mRNA molecules with different Kozak sequences (Figure 3). Five sequences were selected for testing based on the scoring system: **Frequent** (most frequent in transcriptome), **Rare** (rarest in transcriptome), **Worst** (not present in transcriptome), **Middle** score, and the canonical Kozak sequence. The five translational start sites were directly fused to the second codon of eGFP using a 2-step PCR-based cloning technique, and the DNA transcribed *in* *vitro*. The goal was to assess efficiency of eGFP expression via fluorometry. One-cell embryos were injected with one of the 5 different versions of mRNA. At 24 hours, embryos were collected and homogenized. In order to control for variations in mRNA injection levels and total cell content of homogenized tissue, eGFP fluorometry measurements (using a Qubit fluorometer) were standardized using total protein as well as to injected mRNA levels as determined by qRT-PCR (using *elf1a* as an endogenous normalization control). An expression score was then calculated for each sequence (Figure 4). The highest comparative score correlated to the most efficient expression and the lowest to the least efficient. Notably, the zebrafish frequency-optimized transcription initiation sequence showed a nearly 2-fold increase in fluorescence over the canonical Kozak sequence.

## Discussion

Translation initiation is a critical step in the regulation of gene expression. Hence, it is not surprising that translation initiation would itself be regulated across the genome in a species-specific manner. Yet, the variability of the translation start site sequence among different species has not been fully studied, despite the fact that the canonical Kozak sequence has been extensively used in molecular biology to drive gene expression in a variety of backgrounds and genetic models. Combining analysis of accessible sequence data with molecular biology experiments, we were able to create a species-specific optimal Kozak sequence that was significantly more efficient than the non-species specific canonical Kozak sequence.

Our analysis validates many of the conclusions made in the original publication by Kozak [4]. The conservation of the −3 and +4 nucleotides seems ubiquitous across vertebrates. However, slight changes can be seen as species diverged. The difficulty with a purely bioinformatic analysis is determining what, if any, applies *in* *vivo* to any particular model system. Utilizing zebrafish as a proof of principle, we correlate the bioinformatics predictions with direct measurements of translational efficiency. Although there are inherent biases in the frequency based scoring system that we used, it nevertheless provided a framework for selecting test sequences and identifying trends in distribution. The score system also demonstrated the potential for further analysis due to the size and completeness of each data set.

Of particular note, the similarity between the original (i.e. canonical) Kozak sequence and the most frequent zebrafish consensus sequence would, on its face, suggest fairly similar translation efficiency. Contrary to this, we find an almost 2-fold improvement in translational efficiency between the most frequent zebrafish sequence and the canonical Kozak sequence. Therefore, we must conclude that the canonical Kozak sequence is a poor predictor of translation efficiency in different model organisms. Our results also suggest that studies that struggle from low expression levels might benefit from identifying and utilizing an optimized Kozak sequence. Furthermore, the exquisite sensitivity of gene expression to even slight alterations in the Kozak sequence (especially at the 5′ end), and the inter-species variability, suggest important topics for further study of gene expression regulation. Finally, zebrafish researchers studying transgene expression in embryos may wish to utilize our fluorometer-based assay, which provided rapid and consistent results in a sensitive and highly quantifiable manner.

The relative conservation of a purine at position +4 (Figure 1) leads to a bias in the second amino acid of expressed proteins. Codons that begin in pyrimidines encode the amino acids proline, histidine, glutamine, argenine, phenylalanine, tyrosine, cysteine, and tryptophan (as well as leucine and serine), in addition to serving as STOP codons. By selecting for purines in position +4, nature seems to have selected against the above-mentioned amino acids, while reducing the likelihood of a second codon mutation that would lead to a STOP. Of the codons being selected against, it is interesting to note the preponderance of large and/or charged amino acids.

Our approach, in principle, can be applied to other model systems. For example, in a culture-based system, this assay could rapidly test a plethora of start sites to determine the contribution of local nucleotide combinations to translation efficiency. Such data may facilitate the development of gene therapy tools. Our data suggest that changing only a few nucleotides between the “Frequent,” “Rare” and “Worst” sequences could result in a 1–2 orders of magnitude change in expression levels. Depending on protein function, such alterations in expression levels may profoundly impact phenotypes, leading to the phenotypic variability of disease states and providing opportunities for targeted therapies. Additional studies in other organisms would be helpful in testing our predictions.

## Supporting Information

Figure S1 **Validation of RNA injection.** A. qRT-PCR cQ for the control RNA elf1a. B. qRT-PCR cQ for the experimentally-injected RNA encoding eGFP. The “control” bar is from an uninjected control. C. The raw data for A and B, representing 4 groups of 25 embryos for each condition. D. Gel electrophoresis of the RNA that was injected, showing very similar amounts per loaded volume.
(TIF)

List S1 **Ranking list of 250 translation initiation consensus sequences from zebrafish genome.** Higher sequence scores indicate increased selection for individual nucleotides within the sequence.
(XLSX)

## References

[1] Crick F (1970) Central dogma of molecular biology. Nature 227(5258): 561–3. 4913914 10.1038/227561a0

[2] Shine J, Dalgarno L (1974) The 3′-Terminal Sequence of *Escherichia coli* 16S Ribosomal RNA: Complementarity to Nonsense Triplets and Ribosome Binding Sites. PNAS 71(4): 1342–1346. 4598299 10.1073/pnas.71.4.1342 PMC388224

[3] Sachs AB, Sarnow P, Hentze MW (1997) Starting at the Beginning, Middle, and End: Translation Initiation in Eukaryotes. Cell 89: 831–838. 9200601 10.1016/s0092-8674(00)80268-8

[4] Kozak M (1987) An analysis of 5′ noncoding sequences from 699 vertebrate messenger RNAs. Nucleic Acids Res. 15(20): 8125–48. 10.1093/nar/15.20.8125 PMC306349 3313277

[5] Kimmel CB, Ballard WW, Kimmel SR, Ullmann B, Schilling TF (1995) Developmental Dynamics. 203: 253–310. 10.1002/aja.1002030302 8589427

[6] Peterson SM, Freeman JL (2009) RNA Isolation From Embryonic Zebrafish and cDNA Synthesis for Gene Expression Analysis. JoVE doi:10.3791/1470. 10.3791/1470 PMC3152201 19684565

[7] Swindell WR, Johnston A, Sun L, Xing X, Fisher GJ, et al. (2012). Mouse and Human and an Unexpectedly Decreased Inflammatory Signature. PLOSone doi:10.1371/journal.pone.0033204. 10.1371/journal.pone.0033204 PMC3296693 22413003

