# Supplementary figures and images for: Natural Variability of Kozak Sequences Correlates with Function in a Zebrafish Model
Source: PLoS One. 2014 Sep 23;9(9):e108475. doi: 10.1371/journal.pone.0108475 (PMC4172775; doi:10.1371/journal.pone.0108475)

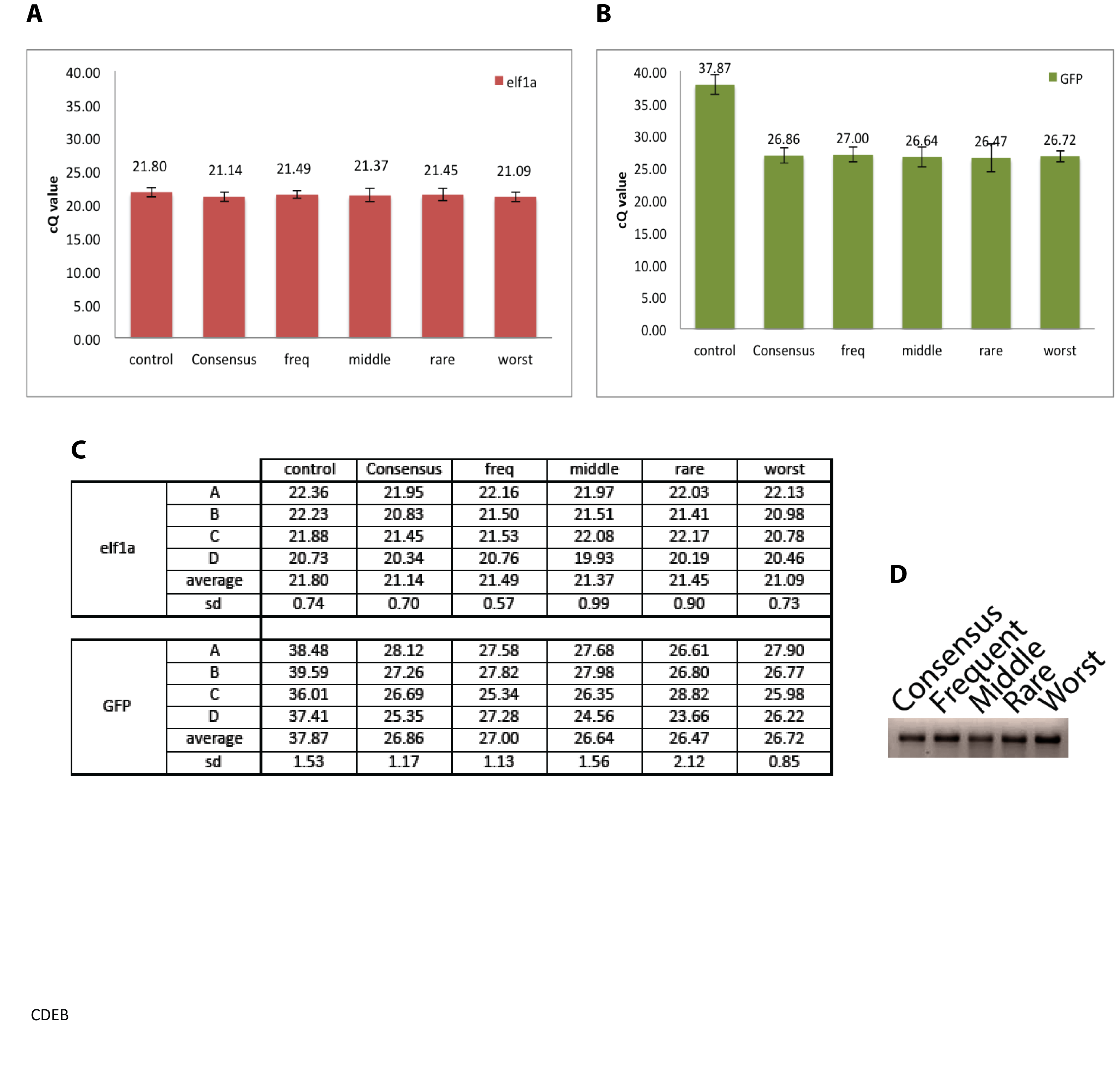

Supplement: Figure S1 — Validation of RNA injection. A. qRT-PCR cQ for the control RNA elf1a. B. qRT-PCR cQ for the experimentally-injected RNA encoding eGFP. The “control” bar is from an uninjected control. C. The raw data for A and B, representing 4 groups of 25 embryos for each condition. D. Gel electrophoresis of the RNA that was injected, showing very similar amounts per loaded volume. (TIF) [file pone.0108475.s001.tif]
